# A Multimedia Educational Module for Teaching Early Medical Neuroanatomy

**DOI:** 10.15766/mep_2374-8265.10885

**Published:** 2020-03-06

**Authors:** Matthew C. Welch, Jonathan Yu, M. Benjamin Larkin, Erin K. Graves, David Mears

**Affiliations:** 1 Medical Student, Uniformed Services University of the Health Sciences F. Edward Hébert School of Medicine; 2 Neurosurgery Resident, Department of Neurosurgery, Baylor College of Medicine; 3 Neurosurgery Resident, Department of Neurosurgery, Temple University; 4 Associate Professor, Department of Anatomy, Physiology, and Genetics, Uniformed Services University of the Health Sciences F. Edward Hébert School of Medicine

**Keywords:** Neuroanatomy, Neurophobia, Dissection, Cadaver, Anatomy, Curriculum, Gross Anatomy, Laboratory Education, Neuroscience, Neurosurgery

## Abstract

**Introduction:**

As the global burden of neurological disorders continues to rise, physicians’ need for a solid understanding of neuroanatomy is becoming more important. Traditional neuroanatomy curricula offer a limited approach to educating a diverse profile of learning styles. In an attempt to incorporate recent literature addressing diverse learning formats, we developed and evaluated two new image-based resources for the neuroscience curriculum.

**Methods:**

We created narrated videos demonstrating the brain dissections that students were to perform in the laboratory and quiz-style, postdissection review slides for later self-guided study. These were offered as optional study aids to two classes of preclerkship medical students at the Uniformed Services University of the Health Sciences F. Edward Hébert School of Medicine. Effectiveness was evaluated through examination questions, and a survey was administered to one of the classes to assess usage of and satisfaction with the materials.

**Results:**

Mean scores on the practical examination questions were 83% and 89% for the two classes of students given the resources. Notably, 100% of respondents used the review slides after the laboratory, and more than 99% found them very helpful or extremely helpful for learning relevant concepts.

**Discussion:**

Our results support the usefulness of these resources as learning tools for neuroanatomy. These resources were meant to augment various traditional resources (textbooks, lecture) to provide a broad range of study options in line with current research. Our experience suggests that similar tools could be developed for application in other visually based content areas of the preclerkship curriculum.

## Educational Objectives

By the end of this educational module, students will be able to:

1. Apply neuroanatomy concepts in laboratory dissection exercises.
2. Attain a three-dimensional understanding of significant anatomical relationships.
3. Draw clinical correlations from neuroanatomy.

## Introduction

In the early 1990s, Ralph Jozefowicz, a physician dual-certified in internal medicine and neurology, observed a highly prevalent condition among his medical students that he described as “a fear of the neural sciences and clinical neurology.”^1^ In 1994, he published an article on the affliction, proposing that the condition is secondary to students’ perception that the neural sciences and clinical neurology are excessively complex. Dr. Jozefowicz dubbed this condition *neurophobia*, and a PubMed search for the term reveals that to date, nearly 60 more works have been published on the topic. Neuroanatomy, a subset of the neural sciences known to invoke neurophobia, seems to induce fear and disdain among medical professionals across all levels, from first-year medical students through attendings in nonneurological specialties.^2,3^ The global burden of neurological disease is rising rapidly.^4^ Therefore, a solid foundation in neuroanatomy has broad clinical implications across many specialties, and a generalized understanding of neuroanatomy by primary care physicians can be paramount.^2^ In an acute setting, understanding the clinical presentation of peripheral versus central facial palsy is crucial to ensuring appropriate diagnosis, imaging, and treatment. Perhaps more relevant to both Dr. Jozefowicz's students and our own, a better example might be the question of why increased intracranial pressure could cause an oculomotor palsy, an understanding of which ties in neuroanatomy, clinical neurology, and appropriate pharmacology. Unfortunately, studies have suggested that even for students who succeed in the classroom setting, the retention of this knowledge beyond medical school is minimal.^5^

The study of human anatomy has been documented for thousands of years; however, the past few decades have witnessed new didactic modalities and techniques developing at an unprecedented rate.^2,6^ With the introduction of electronic resources and the ubiquitous nature of internet access, the classical stereotype of a professor speaking at length to a large classroom of students feverishly scribbling notes is waning in medical education.^2^ Multiple studies have revealed students’ preferences for a breadth of resources, self-directed study, and electronic integration.^6–9^ With this in mind, we set out to develop learning resources to supplement the traditional lecture-based and hands-on laboratory teaching that has been the staple in neuroanatomy education for decades. The materials have been designed to be implemented in a self-paced, independent learning format. The target audience is preclerkship medical students or students in other health professions curricula who are at the beginning their neuroanatomy education. Students should be familiar with basic anatomical terminology and should have appropriate training in biological tissue handling and laboratory safety.

Several learning tools for brain anatomy have been described and made available in *MedEdPORTAL* within the past few years. These include two sets of instructions for making three-dimensional brain dissections^10,11^; a team-based learning activity that focuses on key brain structures and functions^12^; and a rotatable, sliceable, stereoscopic “virtual brain.”^13^ Although there is some overlap between our learning module and these previous works, our module is broader in scope, as it covers both the external and internal features of the brain, as well as the brainstem and cranial nerves. Our work is also unique in that it combines multiple learning formats, comprising a hands-on, wet lab with written instructions and video demonstration; a self-paced, interactive review of structure, function, and clinical application; and ample questions for formative assessment. This combination provides repetition to help reinforce concepts while also affording learners a substantial degree of individual control over the learning experience. Finally, this project adds to the educational literature by providing insights into medical students’ usage of and preference for the individual tools within a multimedia learning module. This information can be used by educators to guide the implementation of this package at their institutions or the design of similar packages for other content areas in the preclerkship curriculum.

## Methods

### Development of the Learning Module

We developed this learning activity for use in the preclerkship neuroscience module in the integrated, systems-based medical curriculum at the Uniformed Services University of the Health Sciences (USU). The project was motivated by frequent requests from students for additional visual-based learning aids to supplement traditional lecture and laboratory exercises since the new curriculum was implemented at USU in 2012. In response to these requests and in an attempt to incorporate the student preferences examined by Prithishkumar and Michael,^8^ we produced both anatomical dissection videos (Appendices E and F) and quiz-style anatomy review slides (Appendices G and H). These tools were intended to assist students’ understanding of neuroanatomy in three ways: (1) guiding the students through the steps of the actual dissection to eliminate concerns associated with the logistics of dissection, (2) focusing their attention on the learning objectives developed by the teaching staff, and (3) utilizing clinical correlation as appropriate to retain interest and foster understanding of application. The videos, which demonstrated each step that students were expected to take once they entered the anatomy laboratory, were envisioned as being particularly helpful to prepare the students to perform the dissections and therefore learn more efficiently during the laboratory session. The postdissection review slides were developed primarily for use after the lab to aid understanding. We chose the content of these resources to match that of the introductory lectures and laboratory exercises in the neuroscience module. This content was selected by the neuroscience faculty and has been reviewed annually by both the faculty and the Executive Curriculum Committee.

### Suggested Use of the Module

To fulfill all of the expected learning objectives, the provided resource files and associated documents (Appendices A–I) should be considered as a complete package rather than as stand-alone components and, as such, should be used all-inclusively. The following items are necessary for each group of four students to complete the brain dissections:

• Anatomy textbooks^14,15^: The referenced textbooks were used for suggested reading (see Appendix B) to provide relevant clinical associations and further highlight the anatomy being taught in the module.
• One cadaveric brain (in cases where human brains cannot be purchased for logistical or regulatory reasons, another option is to use brains that are removed from human cadavers during a gross anatomy course, as we have described previously^16^).
• One red biohazard bag for collection of dissection products.
• Personal protective equipment: nitrile gloves, eye protection, scrubs, lab coats/aprons, and face masks.
• Tools for dissection (see Appendices C and D, final page).

### Implementation of the Learning Module

This neuroanatomy learning module was used by two classes of medical students as part of their larger preclerkship neuroscience module (354 students total). The activity occurred on the first 2 days of the module. All materials were available to first-year medical students at the beginning of their neuroscience module and could be used at the discretion of the student. Prior to the dissection, we encouraged students to study the material provided to them (see Appendices C–H) to maximize dissection preparation. We also gave the students an optional predissection questionnaires (Appendix A) to assess their current level of knowledge, a list of suggested readings, and written step-by-step instructions with the list of structures to be identified during the dissections (Appendices C and D). In addition, we instructed students to watch the video tutorials (Appendices E and F). Once the dissections were completed, we encouraged students to study the relevant postdissection review slides (Appendices G and H). The review slides reinforced the identification of checklisted structures and associated key anatomical relationships. They also featured practice questions to apply learned concepts from the suggested readings, video tutorials, and anatomy dissection. Please note that editing these files will disrupt the internal links within the animations of the review slide presentation, and therefore, editing is strongly discouraged.

### Logistics

We gave each group of four students a single preserved human brain to carry out both the external and internal topography labs. We stored the brains in thymol solution, and they were available for students to use within the anatomy lab both during and after the laboratory session. Setup and cleanup time for the two laboratory sessions varied depending on the availability of faculty and the group's own pace of work and generally ranged from 1.5 to 2 hours per lab.

### Data Gathering

To evaluate students’ understanding after completing the learning module and performing the dissections, we administered a practical examination consisting of 11 questions drawn from the content of the activity. Following the practical examination, we asked the students in one class to voluntarily complete a written, 5-point Likert-scale survey. The surveys were given on paper during a mandatory laboratory, and participation was optional. We created the survey to assess students' usage of the learning tools, as well as their opinions of the helpfulness of these tools for preparing to perform the laboratory and learning the anatomy in preparation for the practical examination. Survey responses were intentionally not correlated to anatomy examination scores in the interest of students’ privacy and to ensure honesty in their responses.

## Results

The mean scores for the practical examination for the two classes of students who were given the learning resources were 83% and 89% (standard deviations were 11% and 10%, respectively).

For the class that was given the usage and satisfaction questionnaire, 160 students (89%) completed the external topography survey, and 154 students (86%) completed the internal topography survey. As shown in Tables 1 and 2, almost all students who used the videos found them helpful, giving ratings of 3 out of 5 or higher when asked, “How helpful was the video in preparing you to perform the dissection?” and “How helpful was the video as an aid for learning the relevant anatomy?” (3 = *somewhat helpful,* 4 = *very helpful,* 5 = *extremely helpful*). Fifty-five percent of respondents (*n* = 57) rated the external topography video as very helpful or extremely helpful for preparing to perform the dissection. The percentage of students rating the internal topography video as very helpful or extremely helpful for dissection preparation was slightly smaller (48%, *n* = 45).

**Table 1.** Learner Feedback for External Topography Learning Tools (*N* = 160, 0.9% Item Omission Rate)

	Response^a^ (%)				
Question	1	2	3	4	5
How helpful was the video in preparing you to perform the dissection?	0.0	3.9	40.8	38.8	16.5
How helpful was the video as an aid for learning the relevant anatomy?	0.0	7.8	45.6	33.0	13.6
How helpful were the review slides in preparing you to perform the dissection?	0.0	0.7	8.6	12.2	78.5
How helpful were the review slides as an aid for learning the relevant anatomy?	0.0	0.0	0.0	1.8	98.2
How helpful was the cadaveric brain as an aid for learning the relevant anatomy? (The brain was not available before the dissection.)	1.3	2.6	37.4	36.8	21.9

a Rated on a 5-point Likert scale (1 = *not helpful at all,* 5 = *extremely helpful*).

**Table 2.** Learner Feedback for Internal Topography Learning Tools (*N* = 154, 0.3% Item Omission Rate)

	Response^a^ (%)				
Question	1	2	3	4	5
How helpful was the video in preparing you to perform the dissection?	0.0	5.4	46.2	35.5	12.9
How helpful was the video as an aid for learning the relevant anatomy?	0.0	7.3	47.9	32.3	12.5
How helpful were the review slides in preparing you to perform the dissection?	0.0	0.0	9.9	15.2	74.9
How helpful were the review slides as an aid for learning the relevant anatomy?	0.0	0.1	0.0	3.2	96.7
How helpful was the cadaveric brain as an aid for learning the relevant anatomy? (The brain was not available before the dissection.)	0.7	2.7	38.9	38.9	18.8

a Rated on a 5-point Likert scale (1 = *not helpful at all,* 5 = *extremely helpful*).

Student reaction to the postdissection review slides was overwhelmingly positive. Table 1 shows that 78% of responding students (*n* = 109) rated the external topography postdissection review slides as extremely helpful for preparing for the dissection, and 98% (*n* = 157) rated them as extremely helpful for learning the anatomy. Table 2 shows a similar positive response for the internal topography review slides, with 75% (*n* = 98) and 96% (*n* = 148) of students providing the highest rating for preparing for the dissection and learning the anatomy, respectively.

We also surveyed the students to assess when and how frequently they used these learning tools. Tables 3 and 4 show that the majority of students who used the videos to prepare for the dissection or study for the examination did so once. Usage of the videos was higher prior to the laboratory sessions than afterward, although interestingly, 40% of respondents (*n* = 63) did not view the external topography video prior to the lab and 44% (*n* = 68) did not view the internal topography video before the dissection. Nearly 80% of respondents used the review slides prior to the corresponding laboratory sessions. After the dissections, 100% of respondents used the review slides, with a majority reporting that they used each of the slide sets more than five times.

**Table 3.** Usage of External Topography Learning Tools (*N* = 160, 0.9% Item Omission Rate)

	Response (%)			
Question	0	1	2–4	≥5
How many times did you view the video prior to the lab dissection?	39.9	54.4	5.7	0.0
How many times did you view the video to study for the exam?	63.2	27.1	8.4	1.3
How many times did you use the review slides prior to the lab dissection?	20.7	44.0	26.4	8.9
How many times did you use the review slides to study for the exam?	0.0	3.1	38.3	58.6
How many times did you use the cadaveric brain after the laboratory session in preparation for the exam? (The brain was not available before the dissection.)	22.1	41.1	34.9	1.9

**Table 4.** Usage of Internal Topography Learning Tools (*N* = 154, 0.3% Item Omission Rate)

	Response (%)			
Question	0	1	2–4	≥5
How many times did you view the video prior to the lab dissection?	44.1	50.0	5.2	0.7
How many times did you view the video after the laboratory session in preparation for the exam?	66.9	26.6	4.6	1.9
How many times did you use the review slides prior to the lab dissection?	21.2	46.3	22.6	9.9
How many times did you use the review slides after the laboratory session in preparation for the exam?	0.0	3.9	40.2	55.9
How many times did you use the cadaveric brain after the laboratory session in preparation for the exam? (The brain was not available before the dissection.)	20.9	45.7	28.7	4.7

The cadaveric brain used for the dissection was also available to students after the laboratory session as a study resource. Tables 3 and 4 show that a majority of students used the cadaveric brain at least once or more for further study (external topography use: 78%, *n* = 123; internal topography use: 79%, *n* = 121). Most students who revisited the brain specimen in preparation for the examination did so once. For both labs, nearly 60% of respondents rated the cadaveric brain as very helpful or extremely helpful for learning the anatomy (Tables 1 and 2).

## Discussion

Neuroanatomy is frequently singled out as one of the most challenging of the anatomy classes offered to early-level medical students.^1–3^ One of the reasons behind this perception, as discussed by Hazelton,^2^ is the “complexity and interconnectedness of the central nervous system,” necessitating that it be taught in a more system-based than topographical manner. Hazelton also points out the inconvenience that central nervous system pathology does not present locally, resulting in the classic “identify the lesion” board question and inducing a disconnect between pathology and anatomy not found in other organ systems, such as musculoskeletal or gastrointestinal anatomy.^3^

Our intentions were to reconcile this difficulty with the fact that a basic understanding of the structure, vasculature, and diseases of the brain and spinal cord are important to physicians in virtually every specialty.^2^ The videos and review slides are intended to work with associated lectures, textbook readings, and, most importantly, laboratory dissections of a medical school–level neuroscience course (see Appendices C and D, as well as textbooks by Agur and Dalley,^14^ Moore, Dalley, and Agur,^15^ Blumenfeld,^17^ and Haines^18^). In the face of a changing medical education paradigm in which cadaveric dissections are becoming more rare,^19^ we believe that the dissections themselves, a cornerstone of medical education for hundreds of years, are the most effective way to understand, retain, and recall knowledge of anatomy.^2,19,20^ Therefore, the videos and postdissection review slides in our resource are intended to synergistically augment the dissections. The combined results of the practical examinations and the usage surveys indicate that the learning resources were effective tools for accomplishing the stated educational objectives.

By far, the most popular resource was the postdissection review slides. Both sets of review slides were used by 100% of respondents in preparation for the examination, and 96% to 99% of students found them extremely helpful for learning the anatomy. The results also suggest that the slides were most well received for reviewing the anatomy after the lab rather than for preparing for the dissection, consistent with the instructors’ intentions. Similarly, the videos received greater use prior to the dissection, in line with their intended purpose as preparatory materials. The videos were very popular among those who watched them; however, more than 40% of survey respondents did not use them prior to the corresponding dissection. This is in contrast to our prior publication in which a follow-up survey revealed that 100% of respondents watched a preparatory video of a cadaveric dissection of the cranial cavity at least once before the laboratory session.^16^ We note several explanations for this apparent disparity—variations in the technical difficulty of the dissections, different questions on the surveys administered to assess the resources, and the fact that the videos for the current study corresponded to the first two dissections of the module—so students may have felt that they had too little time to access all of the preparatory materials. Additionally, some students may simply have preferred not to learn by this modality, some may have preferred more control over the pace of the resource (suggested by the preference toward the review slides and the strong student preference for self-directed study^9,20^), and some may not have studied for this test no matter the resources available.

The adoption of abbreviated preclerkship periods in modern medical curricula has instigated a reduction in instructor-guided learning time.^2^ This new paradigm has created a need for resources to augment traditional classroom-based learning activities; at the same time, students have repeatedly expressed interest in self-directed study.^9,20^ Our results suggest that online resources, such as the videos and review slides described here, support these didactic shifts.

Although not every student used every resource, the breadth of resources made available to students was intended to provide studying options between active and passive, videos and pictures, and so on, as well as to provide ample opportunity for spaced repetition, which is a well-documented and necessary element of adult learning.^21,22^ In light of these goals, we consider the survey results promising; each resource was used by most students in the class, and each proved helpful for its intended use.

### Limitations

As mentioned earlier, we made a determination that the class size and the response rate of our survey were not large enough to sufficiently anonymize the data and establish a correlation between examination grades and studying habits. For that reason, it was not possible to comment on the efficacy of any particular combination of these resources in relation to examination performance. Furthermore, an analysis of similar methods in 2016 in which examination performance was compared to the use of dissection audiovisual resources showed mixed results.^23^ We therefore acknowledge that although our data suggest the students enjoyed the new resources, we are unable to conclude that they developed more robust neuroanatomical understanding as a result of their use.

We also acknowledge the inherent selection bias when a response rate is below 100%. Although the opinions of the 11% or 14% (external topography and internal topography surveys, respectively) of students who did not answer the survey cannot be known, we agree that response rates were high enough to allow discussion of generalized results. Furthermore, to address these limitations, we chose not to propose correlations between use of our resources and long-term (postgraduation) clinical retention of neuroanatomy.

Our conclusions are therefore threefold: that the students seemed to enjoy having the expanded resources, that they were able to learn the material without needing to spend countless hours in the anatomy laboratory, and that this approach contributes to reduced stress levels, a more enjoyable learning experience, and greater enthusiasm for neuroscience as a discipline (which has been suggested to correlate with better retention of the material^3^). We also believe that although it remains to be seen whether these tools help students learn neuroanatomy more effectively, students do learn more efficiently, allowing them more time for further self-directed study.

### Future Direction

The 2008 study by Mateen and D'Eon^5^ showed a drastic loss of basic neurology knowledge from the first year of medical school to the fourth year based on a readministered first-year examination. In an attempt both to remove the subjectivity inherent in the surveys we administered and to better approximate long-term retention, we plan to explore this phenomenon with the first USU class exposed to these neuroanatomy modalities (USU class of 2020) and assess the impact, if any, that the videos and/or review slides have on class members' retention of basic neuroscience and anatomy.

Finally, as of this publication, three videos have been produced along with associated review slides: external topography of the brain; internal topography of the brain; and scalp, meninges, and dural partitions.^16^ Based on continued positive feedback from students and teaching assistants, our intentions are to incorporate this set of resources into each of the 11 dissections in USU's neuroscience module. The survey results show student appreciation for the recent expansion of resources consistent with their intended purposes. This encouragement motivates the continued use and development of e-learning tools to supplement neuroanatomy education, with the ultimate goal of increasing understanding and retention, thereby reducing neurophobia.

## Appendices

A. Predissection Questionnaire.docx
B. References for Predissection Questionnaire.docx
C. External Topography Dissection Instructions.docx
D. Internal Topography Dissection Instructions.docx
E. External Topography Dissection Video Tutorial.mp4
F. Internal Topography Dissection Video Tutorial.mp4
G. External Topography Review Slides.pptx
H. Internal Topography Review Slides.pptx
I. External-Internal Survey.docx All appendices are peer reviewed as integral parts of the Original Publication.

## References

[1] Jozefowicz RF Neurophobia: the fear of neurology among medical students. Arch Neurol. 1994;51(4):328–329. https://doi.org/10.1001/archneur.1994.00540160018003 8155008 10.1001/archneur.1994.00540160018003

[2] Hazelton L Changing concepts of neuroanatomy teaching in medical education. Teach Learn Med. 2011;23(4):359–364. https://doi.org/10.1080/10401334.2011.611777 22004321 10.1080/10401334.2011.611777

[3] Javaid MA, Chakraborty S, Cryan JF, Schellekens H, Toulouse A Understanding neurophobia: reasons behind impaired understanding and learning of neuroanatomy in cross-disciplinary healthcare students. Anat Sci Educ. 2018;11(1):81–93. https://doi.org/10.1002/ase.1711 28628732 10.1002/ase.1711

[4] GBD 2016 Neurology Collaborators. Global, regional, and national burden of neurological disorders, 1990–2016: a systematic analysis for the Global Burden of Disease Study 2016. Lancet Neurol. 2019;18(5):459–480. https://doi.org/10.1016/S1474-4422(18)30499-X 30879893 10.1016/S1474-4422(18)30499-X PMC6459001

[5] Mateen FJ, D'Eon MF Neuroanatomy: a single institution study of knowledge loss. Med Teach. 2008;30(5):537–539. https://doi.org/10.1080/01421590802064880 18677658 10.1080/01421590802064880

[6] Samarakoon L, Fernando T, Rodrigo C, Rajapakse S Learning styles and approaches to learning among medical undergraduates and postgraduates. BMC Med Educ. 2013;13:42 https://doi.org/10.1186/1472-6920-13-42 23521845 10.1186/1472-6920-13-42 PMC3620557

[7] Lujan HL, DiCarlo SE First-year medical students prefer multiple learning styles. Adv Physiol Educ. 2006;30(1):13–16. https://doi.org/10.1152/advan.00045.2005 16481603 10.1152/advan.00045.2005

[8] Prithishkumar IJ, Michael SA Understanding your student: using the VARK model. J Postgrad Med. 2014;60(2):183–186. https://doi.org/10.4103/0022-3859.132337 24823519 10.4103/0022-3859.132337

[9] Choi-Lundberg DL, Low TF, Patman P, Turner P, Sinha SN Medical student preferences for self-directed study resources in gross anatomy. Anat Sci Educ. 2016;9(2):150–160. https://doi.org/10.1002/ase.1549 26033851 10.1002/ase.1549

[10] Rae G, Oliver P, Casey G, Cork R A deep brain dissection protocol. MedEdPORTAL. 2014;10:9855 https://doi.org/10.15766/mep_2374-8265.9855

[11] Detton AJ, Lee LMJ Regional dissection of the human brain. MedEdPORTAL. 2011;7:9016 https://doi.org/10.15766/mep_2374-8265.9016

[12] Barratt D, Stevens C, Smirnoff L Neuroanatomy team-based learning module. MedEdPORTAL. 2015;11:10144 https://doi.org/10.15766/mep_2374-8265.10144

[13] Wiper-Bergeron N, Weber J, Imbeault S, Goodwin S Neuronline. MedEdPORTAL. 2011;7:8266 https://doi.org/10.15766/mep_2374-8265.8266

[14] Agur AMR, Dalley AF Grant's Atlas of Anatomy. 14th ed Philadelphia, PA: Wolters Kluwer; 2017.

[15] Moore KL, Dalley AF, Agur AMR Clinically Oriented Anatomy. 7th ed Philadelphia, PA: Wolters Kluwer; 2014.

[16] Larkin MB, Graves E, Rees R, Mears D A multimedia dissection module for scalp, meninges, and dural partitions. MedEdPORTAL. 2018;14:10695 https://doi.org/10.15766/mep_2374-8265.10695 30800895 10.15766/mep_2374-8265.10695 PMC6342347

[17] Blumenfeld H Neuroanatomy Through Clinical Cases. 2nd ed Sunderland, MA: Sinauer Associates; 2010.

[18] Haines DE Neuroanatomy in Clinical Context: An Atlas of Structures, Sections, Systems, and Syndromes. 9th ed Philadelphia, PA: Wolters Kluwer Health; 2015.

[19] Older J Anatomy: a must for teaching the next generation. Surgeon. 2004;2(2):79–90. https://doi.org/10.1016/S1479-666X(04)80050-7 15568432 10.1016/s1479-666x(04)80050-7

[20] Davis CR, Bates AS, Ellis H, Roberts AM Human anatomy: let the students tell us how to teach. Anat Sci Educ. 2014;7(4):262–272. https://doi.org/10.1002/ase.1424 24249485 10.1002/ase.1424

[21] Kerfoot BP, DeWolf WC, Masser BA, Church PA, Federman DD Spaced education improves the retention of clinical knowledge by medical students: a randomised controlled trial. Med Educ. 2007;41(1):23–31. https://doi.org/10.1111/j.1365-2929.2006.02644.x 17209889 10.1111/j.1365-2929.2006.02644.x

[22] Shaw T, Long A, Chopra S, Kerfoot BP Impact on clinical behavior of face-to-face continuing medical education blended with online spaced education: a randomized controlled trial. J Contin Educ Health Prof. 2011;31(2):103–108. https://doi.org/10.1002/chp.20113 21671276 10.1002/chp.20113

[23] Choi-Lundberg DL, Cuellar WA, Williams AM Online dissection audio-visual resources for human anatomy: undergraduate medical students’ usage and learning outcomes. Anat Sci Educ. 2016;9(6):545–554. https://doi.org/10.1002/ase.1607 27802370 10.1002/ase.1607

