# Supplementary material for: A Multimedia Educational Module for Teaching Early Medical Neuroanatomy
Source: MedEdPORTAL. 2020 Mar 6;16:10885. doi: 10.15766/mep_2374-8265.10885 (PMC7067615; doi:10.15766/mep_2374-8265.10885)
Supplement: Supplementary file 1 — A. Predissection Questionnaire.docx B. References for Predissection Questionnaire.docx C. External Topography Dissection Instructions.docx D. Internal Topography Dissection Instructions.docx E. External Topography Dissection Video Tutorial.mp4 F. Internal Topography Dissection Video Tutorial.mp4 G. External Topography Review Slides.pptx H. Internal Topography Review Slides.pptx I. External-Internal Survey.docx [file mep-16-10885-s001.zip › A. Predissection Questionnaire.docx]

**Pre-Dissection Questionnaire: External Topography of the Brain**

1) Where are the following functional regions of the brain located? (Indicate gyri and lobe)

| **AREA** | **GYRUS or GYRI** | **LOBE** |
| --- | --- | --- |
| Primary motor cortex |  |  |
| Primary somatosensory cortex |  |  |
| Primary visual cortex |  |  |
| Primary auditory cortex |  |  |

2) On which side of the body would neurologic deficits be observed following a lesion of the primary somatosensory cortex?

_____________________________________________________________________________________

3) Where is Broca’s area located?

_____________________________________________________________________________________

4) What is the function of Broca’s area?

_____________________________________________________________________________________

5) What is the function of Wernicke’s area?

_____________________________________________________________________________________

6) How does the aphasia resulting from damage to Broca’s area differ from the aphasia resulting from damage to Wernicke’s area?

_____________________________________________________________________________________

7) What neurological deficit would most likely result from damage to the inferior parietal lobule on the non-dominant side of the brain?

_____________________________________________________________________________________

8) Where is the insula located?

_____________________________________________________________________________________

9) Which part of the body would exhibit motor and sensory deficits in a patient with a lesion of the paracentral lobule?

___________________________________________________________________________________

10) Which gyrus outlines the corpus callosum on the midsagittal surface of the brain?

___________________________________________________________________________________

11) The olfactory bulbs are located on the ventral surface of which lobe of the brain?

___________________________________________________________________________________

12) The uncus is located at the rostral tip of which gyrus?

___________________________________________________________________________________

13) Which cranial nerves attach to the midbrain?

___________________________________________________________________________________

14) Which portion of the brainstem contains the pyramid and the olive?

___________________________________________________________________________________

**Pre-Dissection Questionnaire: Internal Topography of the Brain**

1.	What structure secretes cerebrospinal fluid?

_____________________________________________________________________________________

2.	What structure forms the medial wall of the body of the lateral ventricle?

_____________________________________________________________________________________

3.	Which portion of the ventricular system is located in the midbrain?

_____________________________________________________________________________________

4.	What are the names of the openings that connect the fourth ventricle to the subarachnoid space?

_____________________________________________________________________________________

5.	What fiber bundle crosses the midline just anterior to the column of the fornix?

___________________________________________________________________________________

6.	Which lobe of the brain contains the amygdala and hippocampal formation?

___________________________________________________________________________________

7.	Which structures make up the lentiform nucleus?

_____________________________________________________________________________________

8.	What structure separates the head of the caudate nucleus from the lentiform nucleus?

_____________________________________________________________________________________

9.	What is the arrangement of these internal structures of the brain, from lateral to medial: internal capsule, globus pallidus, putamen, thalamus?

_____________________________________________________________________________________

10.	Which subdivision of the brain contains the substantia nigra?

_____________________________________________________________________________________

**Answers to Pre-Dissection Questionnaire: External Topography of the Brain**

1) Where are the following functional regions of the brain located? (Indicate gyri and lobe)

| **AREA** | **GYRUS or GYRI** | **LOBE** |
| --- | --- | --- |
| Primary motor cortex | PRECENTRAL GYRUS | FRONTAL |
| Primary somatosensory cortex | POSTCENTRAL GYRUS | PARIETAL |
| Primary visual cortex | UPPER AND LOWER BANK OF CALCARINE SULCUS (CUNEUS AND LINGUAL GYRUS) | OCCIPITAL |
| Primary auditory cortex | TRANSVERSE GYRUS OF HESCHL | TEMPORAL |

2)	On which side of the body would neurologic deficits be observed following a lesion of the primary somatosensory cortex? CONTRALATERAL

_____________________________________________________________________________________

3) Where is Broca’s area located? ON THE INFERIOR FRONTAL GYRUS (PARS TRIANGULARIS AND PARS OPERCULARIS) OF THE DOMINANT SIDE OF THE BRAIN (USUALLY LEFT)

_____________________________________________________________________________________

4) What is the function of Broca’s area? MOTOR FUNCTIONS OF LANGUAGE

_____________________________________________________________________________________

5) What is the function of Wernicke’s area? LANGUAGE COMPREHENSION

_____________________________________________________________________________________

6) How does the aphasia resulting from damage to Broca’s area differ from the aphasia resulting from damage to Wernicke’s area? DAMAGE TO BROCA’S AREA CAUSES MOTOR (NON-FLUENT) APHASIA, IN WHICH PATIENTS CAN COMPREHEND LANGUAGE BUT HAVE GREAT DIFFICULTY PRODUCING SPEECH AND WRITING. DAMAGE TO WERNICKE’S AREA PRODUCES FLUENT APHASIA IN WHICH SPEECH IS FLUENT BUT NONSENSICAL AND COMPREHENSION IS IMPAIRED.

_____________________________________________________________________________________

7) What neurological deficit would most likely result from damage to the inferior parietal lobule on the non-dominant side of the brain? CONTRALATERAL HEMI-NEGLECT

_____________________________________________________________________________________

8) Where is the insula located? THE INSULA IS A CORTICAL AREA LOCATED DEEP WITHIN THE LATERAL SULCUS.

_____________________________________________________________________________________

9) Which part of the body would exhibit motor and sensory deficits in a patient with a lesion of the paracentral lobule? THE CONTRALATERAL LOWER LIMB

___________________________________________________________________________________

10) Which gyrus outlines the corpus callosum on the midsagittal surface of the brain? CINGULATE GYRUS

___________________________________________________________________________________

11) The olfactory bulbs are located on the ventral surface of which lobe of the brain? FRONTAL

___________________________________________________________________________________

12) The uncus is located at the rostral tip of which gyrus? PARAHIPPOCAMPAL (IN THE TEMPORAL LOBE)

___________________________________________________________________________________

13) Which cranial nerves attach to the midbrain? (OCULOMOTOR [CN III] AND TROCHLEAR [CN IV])

___________________________________________________________________________________

14. Which portion of the brainstem contains the pyramid and the olive? MEDULLA

___________________________________________________________________________________

**Answers to Pre-Dissection Questionnaire: Internal Topography of the Brain**

1.	What structure secretes cerebrospinal fluid? CHOROID PLEXUS

_____________________________________________________________________________________

2.	What structure forms the medial wall of the body of the lateral ventricle? SEPTUM PELLUCIDUM

_____________________________________________________________________________________

3. Which portion of the ventricular system is located in the midbrain? CEREBRAL AQUEDUCT (AKA AQUEDUCT OF SYLVIUS)

_____________________________________________________________________________________

4. What are the names of the openings that connect the fourth ventricle to the subarachnoid space? (FOREMEN OF MAGENDIE AND FORAMENA OF LUSCHKA)

_____________________________________________________________________________________

5. What fiber bundle crosses the midline just anterior to the column of the fornix? ANTERIOR COMMISSURE

___________________________________________________________________________________

6. Which lobe of the brain contains the amygdala and hippocampal formation? TEMPORAL (THEY ARE LOCATED IN THE MEDIAL TEMPORAL LOBE)

___________________________________________________________________________________

7. Which structures make up the lentiform nucleus? PUTAMEN AND GLOBUS PALLIDUS

_____________________________________________________________________________________

8. What structure separates the head of the caudate nucleus from the lentiform nucleus? ANTERIOR LIMB OF THE INTERNAL CAPSULE

_____________________________________________________________________________________

9. What is the arrangement of these internal structures of the brain, from lateral to medial: internal capsule, globus pallidus, putamen, thalamus? PUTAMEN, GLOBUS PALLIDUS, INTERNAL CAPSULE, THALAMUS

_____________________________________________________________________________________

10. Which subdivision of the brain contains the substantia nigra? MIDBRAIN

_____________________________________________________________________________________
