# Supplementary material for: A Multimedia Educational Module for Teaching Early Medical Neuroanatomy
Source: MedEdPORTAL. 2020 Mar 6;16:10885. doi: 10.15766/mep_2374-8265.10885 (PMC7067615; doi:10.15766/mep_2374-8265.10885)
Supplement: Supplementary file 1 — A. Predissection Questionnaire.docx B. References for Predissection Questionnaire.docx C. External Topography Dissection Instructions.docx D. Internal Topography Dissection Instructions.docx E. External Topography Dissection Video Tutorial.mp4 F. Internal Topography Dissection Video Tutorial.mp4 G. External Topography Review Slides.pptx H. Internal Topography Review Slides.pptx I. External-Internal Survey.docx [file mep-16-10885-s001.zip › B. References for Predissection Questionnaire.docx]

References for Pre-Dissection Questionnaires:

In order to assist students who feel they are having difficulty with the pre-dissection questions during self-learning, the content to answer these questions is covered with in the curriculum material as indicated below. The majority of questions are given in multiple locations with in the curriculum material.

External Topography Questions

1) Suggested Reading: Neuroanatomy through Clinical Cases, 2nd Ed., pp. 28-29; Neuroanatomy in Clinical Context, 9th Ed., p. 13.

2) Suggested Reading: Neuroanatomy through Clinical Cases, 2nd Ed., pp. 28-29; see also post-dissection review slide #2.

3) Suggested Reading: Neuroanatomy through Clinical Cases, 2nd Ed., p. 43; Neuroanatomy in Clinical Context, 9th Ed., p. 12; also see External Topography Video Tutorial (2:55)

4) Suggested Reading: Neuroanatomy through Clinical Cases, 2nd Ed., p. 43; Neuroanatomy in Clinical Context, 9th Ed., p. 12.

5) Suggested Reading: Neuroanatomy through Clinical Cases, 2nd Ed., p. 43; Neuroanatomy in Clinical Context, 9th Ed., p. 12.

6) Suggested Reading: Neuroanatomy through Clinical Cases, 2nd Ed., p. 43; Neuroanatomy in Clinical Context, 9th Ed., p. 12.

7) Suggested Reading: Neuroanatomy through Clinical Cases, 2nd Ed., p. 44.

8) Suggested Reading: Neuroanatomy through Clinical Cases, 2nd Ed., pp. 25, 42; Neuroanatomy in Clinical Context, 9th Ed., p. 36, also see External Topography Video Tutorial (2:10)

9) This is discussed in post-dissection review slide #7 and the Video Tutorial (12:20).

10) Suggested Reading: Neuroanatomy through Clinical Cases, 2nd Ed., pp. 25; see also images on pp. 18 & 20 of Neuroanatomy in Clinical Context, 9th Ed., and post-dissection review slide #7.

11) Suggested Reading: Neuroanatomy through Clinical Cases, 2nd Ed., p. 25, 42; see also Video Tutorial (5:30)

12) See figures in Neuroanatomy through Clinical Cases, 2nd Ed., pp. 27, 42; see also images on pp. 18 & 20 & 26 of Neuroanatomy in Clinical Context, 9th Ed., and post-dissection review slide #5.

13) Suggested Reading: Neuroanatomy through Clinical Cases, 2nd Ed., pp 38-40, see also Video Tutorial (7:55).

14) Suggested Reading: Neuroanatomy through Clinical Cases, 2nd Ed., pp 38-40; Neuroanatomy in Clinical Context, 9th Ed., pp 20, 22, see also Video Tutorial (9:55).

Internal Topography Questions

1) Suggested Reading: Neuroanatomy through Clinical Cases, 2nd Ed., p. 16; see also Internal Topography Video Tutorial (3:25)

2) See images on pp. 78 & 79 of Neuroanatomy in Clinical Context, 9th Ed., and Video Tutorial (1:35)

3) Suggested Reading: Neuroanatomy through Clinical Cases, 2nd Ed., p. 132-136.

4) Suggested Reading: Neuroanatomy through Clinical Cases, 2nd Ed., p. 132-136, see also Video Tutorial (6:40).

5) See images on p. 29 of Neuroanatomy in Clinical Context, 9th Ed., and Video Tutorial (0:35)

6) Suggested Reading: Neuroanatomy through Clinical Cases, 2nd Ed., pp. 41-42;

7) See post-dissection review slide #3, and Video Tutorial (10:25; 25:00).

8) See images on pp. 77, 78, 88 & 89 of Neuroanatomy in Clinical Context, 9th Ed.; see also post-dissection review slides #1,#2, # 8 & #9.

9) See image on pp. 79 of Neuroanatomy in Clinical Context, 9th Ed.; see also post-dissection review slides #3.

10) See Video Tutorial (16:50; 26:20)
