# Supplementary material for: A Multimedia Educational Module for Teaching Early Medical Neuroanatomy
Source: MedEdPORTAL. 2020 Mar 6;16:10885. doi: 10.15766/mep_2374-8265.10885 (PMC7067615; doi:10.15766/mep_2374-8265.10885)
Supplement: Supplementary file 1 — A. Predissection Questionnaire.docx B. References for Predissection Questionnaire.docx C. External Topography Dissection Instructions.docx D. Internal Topography Dissection Instructions.docx E. External Topography Dissection Video Tutorial.mp4 F. Internal Topography Dissection Video Tutorial.mp4 G. External Topography Review Slides.pptx H. Internal Topography Review Slides.pptx I. External-Internal Survey.docx [file mep-16-10885-s001.zip › C. External Topography Dissection Instructions.docx]

**Suggested Reading:**

- Blumenfeld H. *Neuroanatomy through clinical cases*. 2nd ed. Sunderland, MA: Sinauer Associates; 2010:14-17, 24-29, 41-44.
- Haines DE. *Neuroanatomy: an atlas of structures, sections, and systems*. 9^th^ ed. Philadelphia: Lippincott Williams & Wilkins; 2015:11-28, 36.

**Objectives:**

1) Delineate the four lobes of the cerebral hemispheres on a preserved human brain.
2) Identify structures on the lateral, ventral and midsagittal surfaces of the brain.
3) Locate and describe the function of the primary motor cortex, primary somatosensory cortex, primary visual cortex, primary auditory cortex, Broca’s area and Wernicke’s area.
4) Describe the somatotopic organization of the primary motor cortex and primary somatosensory cortex.

**Introduction:**

At the beginning of this dissection, each group will receive a preserved human brain in a bucket containing the preservative thymol. You will use this specimen to study the gross structures of the brain in this and the following laboratory session.

Begin by removing the brain from the bucket and placing it on a white brain board. Carefully remove the arachnoid mater and cerebral blood vessels that course along the surface of the brain. Have an instructor show you where the cranial nerves attach to the brainstem so you can preserve them. Bags will be provided in each bay to dispose of the arachnoid and blood vessels. Discard these materials properly.

Before beginning the lab, refer to Figure 1 and review the directional terminology that will be used extensively throughout the brain dissections.

**Part 1: Lobes of the Cerebral Hemispheres**

**1.1.	Frontal Lobe:**

To delineate the **frontal lobe**, first look for the **central sulcus**. It is located near the middle of the hemisphere and courses vertically downward from the superior edge. Hint: look for two parallel, vertically oriented gyri near the center of the hemisphere. The central sulcus is located between them. The central sulcus ends inferiorly near a deep, more horizontally oriented sulcus, the **lateral sulcus** (a.k.a. **Sylvian fissure**). The frontal lobe is the portion of the hemisphere located rostral (anterior) to the central sulcus and dorsal (superior) to the lateral sulcus.

**1.2.	Parietal Lobe:**

The **parietal lobe** is located behind (caudal to) the frontal lobe. Its rostral limit is the central sulcus. To define its caudal boundary, carefully peer into the **longitudinal fissure** (the deep fissure in the midline) to see the medial aspect of the hemisphere. Toward the caudal end of the medial aspect, find the vertically oriented **parieto-occipital sulcus**. Now draw an imaginary line along the lateral surface of the hemisphere, from the upper end of the parieto-occipital sulcus to the *pre-occipital notch* on the lower edge of the hemisphere (see **Figure 1**). This vertical line marks the parieto-occipital boundary. The inferior border of the parietal lobe is formed by the lateral sulcus (rostrally) and a horizontal extrapolated line drawn from the point where the lateral sulcus bends upward to the midpoint of the vertical extrapolated line previously drawn.

**1.3.	Temporal Lobe:**

The **temporal lobe** is located ventral (inferior) to the frontal and parietal lobes. Its upper border is formed by the lateral sulcus and the horizontal extrapolated line that you made in the previous section. Its caudal boundary is the lower part of the vertical extrapolated line from the previous section, which separates it from the occipital lobe.

**1.4.	Occipital Lobe:**

The **occipital lobe** is located caudal (posterior) to the parietal and temporal lobes, extending to the *occipital pole* at the caudal end of the hemisphere.

**1.5.	Insula:**

Carefully spread apart the lateral sulcus and look for several vertically-oriented gyri within its depths. This is the **insula**, a region of cerebral cortex that was overgrown by the cortex of the frontal, parietal and temporal lobes during development. The parts of these lobes that cover the insula are called the *frontal, parietal* and *temporal opercula*, respectively.

**Part 2: Gyri & Sulci on the Lateral Surface of the Cerebral Hemisphere (Image 2.1)**

**2.1.	Frontal Lobe**

The thick, vertical gyrus located just in front of (rostral to) the central sulcus is the **precentral gyrus**. Notice that it is bordered anteriorly by a vertical sulcus, the *precentral sulcus*, which is oriented parallel to the central sulcus. The precentral gyrus is the location of the **primary motor cortex**, which controls movements of the contralateral side of the body. Indicate the approximate areas that control movements of the face, hand, arm and trunk.

**Where is the motor area for the lower limb?**

Rostral to the precentral gyrus, the lateral aspect of the frontal lobe consists of three horizontally oriented gyri: the **superior frontal gyrus**, the **middle frontal gyrus**, and the **inferior frontal** gyrus. Notice that the inferior frontal gyrus is limited inferiorly by the lateral sulcus.

The inferior frontal gyrus is divided into three parts. The **pars opercularis** is a narrow, vertical gyrus located just rostral to the precentral gyrus. It is part of the covering of the insula. Anterior to pars opercularis is **pars triangularis**, named for its shape. The most rostral portion of the inferior frontal gyrus (rostral to pars triangularis) is **pars orbitale**, so named because this portion of the frontal lobe sits on the roof of the orbit in the cranial cavity.

**What neurological deficit is typically observed following a lesion to broca’s area?**

**Broca’s area** consists of the pars triangularis and pars opercularis of the dominant hemisphere (the left side in most individuals).

**2.2.	Parietal Lobe:**

**What is the function of the postcentral gyrus?**

The vertical gyrus located immediately posterior to the central sulcus is the **postcentral gyrus**. The postcentral gyrus is bordered posteriorly by the *postcentral sulcus*, which is oriented parallel to the central and precentral sulci.

Look for a horizontal sulcus coursing towards the occipital lobe from the postcentral sulcus. This is the *intraparietal* sulcus, which divides the posterior part of the parietal lobe into the **superior parietal lobule** (above the intraparietal sulcus) and the **inferior parietal lobule** (below the intraparietal sulcus). The inferior parietal lobule consists of two gyri shaped like upside-down letter U’s. The **supramarginal gyrus** is the more rostral of the two, and wraps around the posterior end of the lateral sulcus. The **angular gyrus** wraps around the posterior end of the superior temporal sulcus.

**What clinical condition is caused**

**by damage to the inferior parietal lobule on the non-dominant side?**

**2.3.	Temporal Lobe:**

The temporal lobe contains three gyri that course in parallel to the lateral sulcus. These are the **superior temporal gyrus**, the **middle temporal gyrus**, and the **inferior temporal gyrus**. Most or all of the inferior temporal gyrus may be located on the inferior aspect of the hemisphere.

Carefully spread open the lateral sulcus, and look for one or two gryi running transversely (from medial to lateral) on the superior surface of the superior temporal gyrus. These are the **transverse gyri of Heschl**, which represent the primary auditory cortex. Don’t confuse them with the gyri of the insula, which are deeper and more vertically oriented (and not attached to the superior temporal gyrus).

The posterior portion of the superior temporal gyrus of the dominant hemisphere is known as **Wernicke’s area** (some authors also include parts of the angular and supramarginal gyri). Like Broca’s area, it is an important language center. Specifically, it mediates language comprehension.

**How does Wernicke’s aphasia differ from Broca’s aphasia?**

**2.4.	Occipital Lobe:**

The convolutions on the lateral aspect of the occipital lobe are collectively referred to as **occipital gyri**.

**Part 3: Gyri & Sulci on the Ventral Surface of the Cerebral Hemisphere**

**(Image 3.1)**

**3.1.	Frontal Lobe:**

Turn the brain over to look at its ventral surface. Rostrally, you will see the gyri of the ventral surface of the frontal lobe (also called the orbital surface because it sits on the roof of the orbit in the cranial cavity). Find the thin **gyrus rectus** at the medial edge of the ventral surface. The sulcus just lateral to it is the **olfactory sulcus**, so named because the **olfactory bulb** and **olfactory tract** lie within it. The gyri located lateral to the olfactory sulcus are collectively called **orbitofrontal gyri** (or just orbital gyri). At the caudal end of the frontal lobe, find the **lateral sulcus**, which, as on the lateral surface, separates the frontal and temporal lobes.

**3.2.	Temporal Lobe:**

Look for the **collateral sulcus**, a deep sulcus that courses rostro- caudally near the medial edge of the temporal lobe. The gyrus lying medial to it is the **parahippocampal gyrus** (you will probably have to carefully lift up the cerebellar hemisphere to see these medial structures). The wide gyrus (often a double gyrus) located lateral to the collateral sulcus is the **occipitotemporal gyrus.** Lateral to this, find the **inferior temporal gyrus** coursing in the rostro-caudal direction at the lateral edge of the ventral surface of the hemisphere. Remember that you looked for this gyrus on the lateral surface of the temporal lobe, but often it is partly or fully located on the ventral surface.

Follow the parahippocampal gyrus rostrally and find a medially-oriented protrusion near the rostral end. This protrusion is the **uncus**. Notice that it juts out in front of the upper part of the brainstem (the midbrain). You will learn the importance of this relationship in the context of elevated intracranial pressure in several upcoming lectures.

**Part 4: Sub-Telencephalic Structures on the Ventral Surface of the Brain**

**(Image 4.1)**

**4.1. Diencephalon:**

Follow the gyrus rectus and olfactory tracts caudally along the ventral surface of the frontal lobe to find the **optic nerves** (**CN II**). The two optic nerves join at the **optic chiasm**, where some of the fibers of each optic nerve cross to the opposite side. Follow the **optic tracts** caudally from the optic chiasm and see that they wrap around the midbrain, disappearing dorsal to the uncus. The optic nerves, chiasm and tract are all part of the diencephalon.

Proceeding caudally from the optic chiasm, find the **infundibulum**, which connects the hypothalamus to the pituitary gland. Now look behind the infundibulum for two round protrusions, the **mammillary bodies**. The mammillary bodies are part of the hypothalamus and play an important role in memory formation.

**4.2.	Midbrain:**

Look lateral and caudal to the mammillary bodies to find two large, column-like structures, the **crus cerebri,** which are parts of the larger **cerebral peduncles**. Each crus cerebri consists of a massive bundle of nerve fibers that originate in the cerebral cortex and project down to the brain stem and spinal cord. The depression between the two cerebral peduncles is the *interpeduncular fossa*. Two cranial nerves attach to the midbrain. Look for the **oculomotor nerve** (**CN III**) emerging ventrally from the interpeduncular fossa. The **trochlear nerve** (**CN IV**) attaches to the dorsal aspect of the brainstem and then courses around the cerebral peduncle to reach the ventral side. Look for this slender nerve lateral to the cerebral peduncle, near the midbrain-pons junction.

**4.3.	Pons:**

The ventral surface of the pons consists of a prominent bulge, the **basilar pons**. Follow it superiorly and inferiorly to find two horizontal grooves, the *superior and inferior pontine sulci*. These sulci mark the boundary of the pons with the midbrain and medulla, respectively.

The **trigeminal nerve** (**CN V**) attaches to the ventral surface of the pons at the junction of the basilar pons and the middle cerebellar peduncle. The **abducens nerve** (**CN VI**) emerges near the midline at the inferior pontine sulcus. Now look lateral to CN VI in the cerebellopontine angle (region where the pons, medulla and cerebellum all come together) to find the **facial nerve** (**CN VII**) and the **vestibulocochlear nerve** (**CN VIII**). CN VII lies medial to CN VIII, with the slender *intermediate nerve* between them.

**4.4.	Medulla:**

Just lateral to the midline on each side of the medulla is a column-like elevation, the **pyramid**. It is made up of many of the same fibers that were in the crus cerebri. Look for another elevation lateral to the pyramid, the **olive**. Rootlets of the **hypoglossal nerve** (**CN XII**) emerge from the groove between the pyramid and olive (*preolivary sulcus*). Did you notice that CNs III, VI and XII emerge in line with each other near the midline from the midbrain, pons and medulla, respectively? (The formula **3** x 2 = **6** x 2 = **12** can help you remember this relationship!)

Finally, look for rootlets of the **glossopharyngeal** and **vagus nerves** (**CNs IX** & **X**) attaching to the medulla in the groove lateral to the olive (postolivary sulcus). CN IX attaches superior to CN X, but don’t worry about trying to tell them apart.

**Part 5: Hemisected Brain, Midsagittal Surface**	**(Image 5.1)**

Once you have finished identifying structures on the ventral surface of the brain, you should hemisect the brain to look at its midsagittal surface. This is a delicate procedure, so **please wait for an available instructor to help carry it out**. While you are waiting, make sure that you have completely stripped the arachnoid mater and blood vessels from the brain, especially in the midline.

During this dissection, you will identify gyri and sulci on the medial aspect of the cerebral hemisphere. During the next dissection you will go on to identify subcortical structures that can also be seen in this view.

**5.1.	Corpus Callosum:**

The **corpus callosum** (CC) is an excellent landmark on the midsagittal surface of the hemisected brain. Look for this hook-shaped, white structure in the middle of the hemisphere. It is composed of nerve axons that cross the midline to connect the two hemispheres (commissural fibers). Identify its four parts: the **rostrum** (pointed tip located anteriorly and inferiorly); the **genu** (bend at the rostral end of the CC); the **body** (long straight part extending back from the genu); and the **splenium** (rounded caudal end).

**5.2.	Sulci & Gyri on the Medial Aspect of the Cerebral Hemisphere:**

The thick **cingulate gyrus** outlines the corpus callosum on the medial aspect of the hemisphere. Follow it rostrally and observe that it curves in front of the genu and then below (ventral to) the rostrum of the CC. The portion of the cingulate gyrus ventral to the rostrum of the CC is sometimes called the *subcallosal gyrus*. Now follow the cingulate gyrus caudally to see that it thins into an *isthmus* behind and below the splenium of the CC. The isthmus of the cingulate gyrus is continuous with the parahippocampal gyrus of the temporal lobe.

Rostrally, the *cingulate sulcus* separates the cingulate gyrus from the **superior frontal gyrus** (this is the same superior frontal gyrus that you saw on the lateral part of the frontal lobe earlier in the lab). Follow the cingulate sulcus caudally and observe that its caudal end turns upward toward the superior margin of the hemisphere. This upturned portion is called the *marginal sulcus*. The cortical region just rostral (anterior) to the marginal sulcus is the **paracentral lobule**, which represents the primary motor and primary somatosensory area for the lower limb and external genitalia. Look onto the lateral surface of the hemisphere and confirm that the paracentral lobule is indeed the medial continuation of the precentral and postcentral gyri.

You should find two more sulci on the caudal end of the midsagittal surface. The first is the **parieto-occipital sulcus**, which courses obliquely downward from the upper edge of the hemisphere, caudal and somewhat parallel to the marginal sulcus. (You peeked at this one from the other side earlier when you were defining the lobes of the brain, but now you can see it in all its glory). Follow the parieto-occipital sulcus downward and see that it meets up with the **calcarine sulcus,** a very deep sulcus coursing inward from the occipital pole.

The area between the marginal sulcus and the parieto-occipital sulcus is the **precuneus** (part of the parietal lobe) and the triangular region between the parieto-occipital sulcus and the calcarine sulcus is the **cuneus** (part of the occipital lobe). Now look inferior to the calcarine sulcus and find the **lingual gyrus**. Follow it rostrally and see that it is continuous with the parahippocampal gyrus of the temporal lobe. The cortex adjacent to the calcarine sulcus is the **primary visual cortex**. So the primary visual cortex is located partly on the cuneus (upper bank of the calcarine sulcus) and partly on the lingual gyrus (lower bank of the calcarine sulcus).

**Can you describe the visual field defect that results from unilateral damage to the primary visual cortex?**

**Checklist**

**Lateral Aspect of Cerebral Hemisphere:**

Longitudinal fissure

Lateral sulcus (Sylvian Fissure) Central sulcus

Frontal lobe

Parietal lobe

Temporal lobe

Occipital lobe

Insula

Precentral gyrus

Superior frontal gyrus

Middle frontal gyrus

Inferior frontal gyrus

Pars opercularis, triangularis

& orbitale

Broca’s Area

Postcentral gyrus

Superior parietal lobule

Inferior parietal lobule

Supramarginal gyrus

Angular gyrus

Superior temporal gyrus

Middle temporal gyrus

Inferior temporal gyrus

Transverse gyri of Heschl

Wernicke’s area

Occipital gyri

Abducens nerve (CN VI)

Facial nerve (CN VII)

Vestibulocochlear nerve (CNVIII)

Pyramids

Olives

Glossopharyngeal & Vagus nerves (CNs IX & X)

Hypoglossal nerve (CN XII)

Precuneus

Cuneus

Lingual gyrus

Primary visual cortex

**Ventral (Basilar) Aspect of the Whole Brain:**

Gyrus rectus

Olfactory sulcus

Olfactory bulb

Olfactory tract

Orbitofrontal gyri

Lateral sulcus

Inferior temporal gyrus

Occipitotemporal gyrus

Collateral sulcus

Parahippocampal gyrus

Uncus

Optic nerve (CN II)

Optic chiasm

Optic tract

Infundibulum

Mammillary bodies

Crus cerebri (Cerebral peduncles)

Oculomotor nerve (CN III)

Trochlear nerve (CN IV)

Basilar pons

Trigeminal nerve (CN V)

**Midsagittal Aspect of Hemisected Brain:**

Corpus callosum

Rostrum

Genu

Body

Splenium

Cingulate gyrus

Superior frontal gyrus

Paracentral lobule

Parieto-occipital sulcus

Calcarine sulcus

**Images**

**Tools for Dissection:**
