# Supplementary material for: A Multimedia Educational Module for Teaching Early Medical Neuroanatomy
Source: MedEdPORTAL. 2020 Mar 6;16:10885. doi: 10.15766/mep_2374-8265.10885 (PMC7067615; doi:10.15766/mep_2374-8265.10885)
Supplement: Supplementary file 1 — A. Predissection Questionnaire.docx B. References for Predissection Questionnaire.docx C. External Topography Dissection Instructions.docx D. Internal Topography Dissection Instructions.docx E. External Topography Dissection Video Tutorial.mp4 F. Internal Topography Dissection Video Tutorial.mp4 G. External Topography Review Slides.pptx H. Internal Topography Review Slides.pptx I. External-Internal Survey.docx [file mep-16-10885-s001.zip › D. Internal Topography Dissection Instructions.docx]

**Suggested Reading:**

- Blumenfeld H. *Neuroanatomy through clinical cases*. 2nd ed. Sunderland, MA: Sinauer Associates; 2010:740-748
- Haines DE. *Neuroanatomy: an atlas of structures, sections, and systems*. 9^th^ ed. Philadelphia: Lippincott Williams & Wilkins; 2015:28-29; 76-91.

**Objectives:**

1) Identify check-listed structures on the midsagittal aspect of a preserved human brain.
2) Identify check-listed structures on both coronal and axial human brain slices.
3) Demonstrate the anatomical relationships between the major internal structures of the brain, including the thalamus, caudate nucleus, lentiform nucleus, lateral ventricle and internal capsule.
4) Understand the relationships between the lateral ventricle and the following structures: the amygdala, the hippocampal formation, the caudate nucleus, and the fornix.
5) Use the human brain preparation to trace the flow of cerebral spinal fluid (CSF) through the ventricular system.

**Part 1: Subcortical Structures on the Midsagittal Aspect of the Brain**

In the previous brain dissection, you identified the gyri and sulci that are visible on the midsagittal aspect of the hemisected brain, as well as the corpus callosum. Here, you will begin by identifying the remaining structures that can be seen on this view.

**1.1.	Forebrain – Telencephalon & Diencephalon (Image 1.1):**

Review the **corpus callosum** and its four parts: the **rostrum**, **genu**, **body**, and **splenium**. Another important landmark on the midsagittal view is the **thalamus** (dorsal thalamus), an egg-shaped mass located rostral to the splenium and ventral to the body of the corpus callosum. The thalamus is the largest component of the diencephalon. With these two landmarks identified, you’re ready to look for the rest of the structures on the midsagittal aspect of the forebrain.

The **septum pellucidum** is a membrane that extends downward from the body of the corpus callosum. It forms the medial wall of the lateral ventricle, which you’ll explore in a moment. Follow the septum pellucidum ventrally and notice that its most rostral part attaches to the rostrum of the corpus callosum. More caudally it attaches to the **fornix**, a thick fiber bundle that arches over the thalamus and then curves down in front of it. The part of the fornix located dorsal to the thalamus is the **body of the fornix**, and the part located rostral to the thalamus is the **column of the fornix**. The column of the fornix passes behind (caudal to) the **anterior commissure,** a fiber bundle that connects the two temporal lobes. The fibers of the anterior commissure get cut in cross section as they cross the midline just rostral to the column of the fornix. Now look for **lamina terminalis**, a thin membrane that extends ventrally from the anterior commissure down to the optic chiasm at the ventral surface of the diencephalon. Lamina terminalis marks the site of closure of the *anterior neuropore*.

If the septum pellucidum was partially torn on one or both sides when the brain was hemisected, you will be able to see a large space located just lateral to it. This is the **lateral ventricle**, the portion of the ventricular system associated with the telencephalon (cerebral hemisphere). If the septum is intact on both sides, cut a hole in the rostral part of it on one side to look into the lateral ventricle. The **anterior horn of the lateral ventricle** is the part located rostral to the thalamus, and the **body of the lateral ventricle** is the part located dorsal to the thalamus. (Are you starting to appreciate why the thalamus is an important landmark?) The remaining portions of the lateral ventricle (the trigone, inferior horn and posterior horn) are located deeper within the hemisphere, and cannot be seen on the midsagittal view, even if the septum pellucidum has been removed.

Look into the anterior horn of the lateral ventricle and see (or stick your finger in and feel) a large structure bulging into its lateral wall. This is the caudate nucleus. This C-shaped structure follows the shape of the lateral ventricle and has several named subparts. The part rostral to the thalamus (in the lateral wall of the anterior horn of the lateral ventricle) is the **head of the caudate.** The part located dorsal to the thalamus (in the lateral wall of the body of the lateral ventricle) is the **body of the caudate**. The tail of the caudate curves down into the temporal lobe and is only visible in slices. Look for **choroid plexus** (a red, stringy mass) in the floor of the body of the lateral ventricle.

**What is the function of the choroid plexus?**

The lateral ventricle connects to the **third ventricle** (the portion of the ventricular system associated with the diencephalon) via the **interventricular foramen** (**IVF**), also known as the *foramen of Monro*. This opening is located behind the column of the fornix (between the column of the fornix and the thalamus). The third ventricle is the unpaired space in the midline between the left and right diencephalon. It gets cut down the middle when the brain is sectioned (**Image 1.2**).

Relocate the **thalamus** in the dorsolateral wall of the third ventricle. In about 80% of brains, the thalami are connected across the midline by a collection of neural tissue called **massa intermedia** (a.k.a **thalamic adhesion**). Look for it near the center of the thalamus. The triangular area ventral to the thalamus is the **hypothalamus**. Notice that these two structures are separated by a shallow groove in the wall of the third ventricle, the *hypothalamic sulcus*.

On the ventral aspect of the diencephalon, find (from rostral to caudal) the **optic chiasm**, **infundibulum** and **mammillary body**. You saw these same three diencephalic structures on the ventral surface of the brain in the External Topography of the Brain dissection.

Now look for the **pineal body**, a pine cone-shaped gland that attaches to the dorsal aspect of the thalamus and hangs down over the back of the midbrain. It may not be attached after the hemisection. Just ventral to where the pineal body attaches to the thalamus, find the **posterior commissure**, a curved fiber bundle cut in cross section. It is much smaller than the anterior commissure.

**1.2.	Brainstem & Cerebellum (Image 1.3):**

The midbrain is the most rostral division of the brainstem. Dorsally, it contains a roof (tectum) made up of the **superior and inferior colliculi**. Ventral to the tectum, identify the **cerebral aqueduct** (the portion of the ventricular system associated with the midbrain), and ventral to that, the **midbrain tegmentum**. The most ventral structure in the midbrain is the *crus cerebri* (cerebral peduncle), but this structure lies lateral to the midline and is not easy to see on the hemisected brain.

The pons consists of a large ventral region, the bulging **basilar pons**, and a smaller dorsal region, the **pontine tegmentum**, which is continuous with the midbrain tegmentum. Dorsally, the diamond-shaped **fourth ventricle** separates the pons from the **cerebellum**. The roof of the fourth ventricle in the midline is formed by a thin membrane, the **superior (anterior) medullary velum**, which stretches in front of the superior surface of the cerebellum to just below the inferior colliculus.

Now go to the **medulla**. The caudal part of the **fourth ventricle** lies dorsal to the upper medulla. At its caudal limit the fourth ventricle meets the opening of the narrow, internally located central canal of the lower medulla and spinal cord. The fourth ventricle also connects to the subarachnoid space surrounding the brain via the *paired foramina of Luschka* and the unpaired *foramen of Magendie*.

**Part 2: Coronal Brain Slices (Figure 1)**

Before making the slices, use a scalpel to remove the brainstem and cerebellum. Make the cut transversely along a line from the superior pontine sulcus (just above the basilar pons) to just below the inferior colliculus (Figure 1A).

Place the brain on a white brain board and use a large brain knife to make the slices. Be careful, as the knives are very sharp. You will make six coronal slices. Use the drawing in Fig. 1B as well as the anatomical descriptions of each slice as a guide for where to cut. The anatomical descriptions are the headings for each slice in the text below and they tell you the landmarks on the midsagittal surface that should be used for each slice. Each slice should be made in one continuous motion: avoid slicing back and forth or starting and stopping mid-slice, as this can decrease the quality of your slices.

When studying the individual slices, start by placing them with the rostral surface up, as this will allow them to best match the illustrations in the Haines Atlas and the video dissection tutorial. In some cases, the check-listed structures may be better demonstrated on the caudal surface, whether due to the inherent variation of individual brains or the slicing technique, so don’t be afraid to look there as well.

**2.1.	Coronal slice through body & rostrum of the corpus callosum (Slice A; Image 2.1):**

Once you get comfortable with the 3-D layout of the brain, you will probably find it most convenient to start by identifying the large “core” structures at the center of the slice and then use them as landmarks to identify other structures. However, for the first few slices let’s start with the medial and lateral surfaces, which should be familiar to you from your study of the intact hemisphere. In fact, it will be helpful to have the intact hemisphere available as a reference.

Lateral Aspect: Remember from the previous dissection that the lateral surface of the hemisphere consists entirely of cerebral cortex. Place the slice rostral-side up and verify that its entire lateral aspect is lined by a lamina of gray matter, the cerebral cortex. Also, remember that the lateral fissure separates the frontal lobe from the temporal lobe on the lateral aspect of the hemisphere. So, go to the lateral aspect of the slice and find the deep **lateral fissure** separating the frontal lobe, above, from the temporal lobe, below. Follow the lateral fissure medially and see that it ends at the cortex of the **insula**. (This should also bring back memories from the External Topography dissection, when you had to look deep into the lateral fissure to see the insula, which is covered by portions of the frontal, temporal and parietal lobes).

Medial Aspect**:** The structures on the medial edge of the slice are also familiar to you from your study of the hemisected brain. Superiorly, the medial aspect of the slice consists of the cerebral cortex of the frontal lobe. Follow the cortex downward to find a thick, white structure, the **body of the corpus callosum**. From Dissection #1 you know that the gyrus just dorsal to the body of the corpus callosum is the **cingulate gyrus**. The **septum pellucidum** hangs down from the body of the corpus callosum in the midline and attaches ventrally to the *rostrum of the corpus callosum*. The space lateral to the septum pellucidum and ventral to the body of the corpus callosum is the **anterior horn of the lateral ventricle**. By definition, the anterior horn is that portion of the lateral ventricle located rostral to the thalamus and since this slice was made rostral to the thalamus, you must be looking at the anterior horn. Pick up the rostral end of the hemisphere to see the rostral tip of the anterior horn. Notice that it ends medial to the genu of the corpus callosum in the frontal lobe.

Internal Structures**:** Go back to the slice. The large, dark (gray matter) structure located lateral to the anterior horn of the lateral ventricle is the **head of the caudate nucleus**. Remember when you stuck your finger into the anterior horn earlier in this lab to feel the bulging head of the caudate in its lateral wall? If you know that relationship you will always know to look for the head of the caudate lateral to the anterior horn in a 2-D brain slice or image. Lateral and ventral to the head of the caudate is another large gray matter structure, the **putamen**, which is part of the lentiform nucleus. Notice that the putamen and caudate are separated dorsally by a white structure, the **anterior limb of the internal capsule**. At this level the caudate and putamen merge ventrally (below the anterior limb of the internal capsule). The region where these two structures merge is called the nucleus accumbens, which is important in the reward pathway of the brain.

Finally, look lateral to the putamen and observe that it is separated from the **insula** by alternating bands of white and gray matter. From medial to lateral, these are the **external** **capsule** (white matter), **claustrum** (gray matter) and **extreme** **capsule** (white matter). These structures can be helpful as landmarks because you will see them on many slices, always separating the putamen from the insula.

**2.2.	Coronal slice through the anterior commissure (Slice B; Image 2.2):**

Lateral Aspect: As always, the entire lateral surface of the slice is lined with cerebral cortex. Find the **lateral fissure** separating the frontal and temporal lobes, and the **insula** at the medial end of the lateral fissure.

Medial Aspect: On the medial side of the slice, follow the cortex of the frontal lobe downward from the top to find the **cingulate gyrus** and the **body of the corpus callosum** just ventral to it. The **septum pellucidum** extends ventrally from the body of the corpus callosum, but at this level it attaches to the **column of the fornix**. Look at the intact hemisphere and verify that the septum pellucidum attaches to the rostrum of the corpus callosum rostrally (the level of the previous slice) and to the fornix more ventrally (the level of this slice). Just below the column of the fornix find the **anterior commissure**, a band of white fibers extending from the midline downward and laterally toward the temporal lobe. Remember that the column of the fornix passes just behind the anterior commissure on the medial aspect of the hemisphere, so when you see one on a slice you are sure to see the other (go back on the intact hemisphere and review this nice relationship).

Internal Structures: This slice contains many of the same structures seen on the previous slice. Find the **anterior horn of the lateral ventricle** and the **head of the caudate** making up its lateral wall. Again, the **anterior limb of the internal capsule** is located lateral to the head of the caudate and separates it from the **lentiform nucleus**. In this slice, you should be able to see both of the nuclei that make up the lentiform nucleus. The larger, more laterally located nucleus is the **putamen**, and the smaller, wedge-shaped nucleus located medial to the putamen is the **globus pallidus**. As on the previous slice, the putamen is separated from the insula by three thin bands: the **external** **capsule**, **claustrum** and the **extreme** **capsule**.

Temporal Lobe: Finally, look at the medial aspect of the temporal lobe for an almond-shaped nucleus, the **amygdala**. The amygdala is located within the uncus, the medial protrusion at the rostral end of the parahippocampal gyrus that you saw in Dissection #1. Verify on the intact hemisphere that a coronal slice through the anterior commissure also traverses the uncus, so when you see the anterior commissure on a coronal slice, you should expect to see the amygdala in the temporal lobe.

**2.3.	Coronal slice through anterior thalamus & mammillary bodies (Slice C; Image 2.3):**

The big difference between this slice and the previous one is that this one contains the **thalamus**. Look for it on the medial aspect of the slice, medial to the internal capsule and ventral to the lateral ventricle and caudate. Because this slice is at the level of the thalamus, it goes through the **body of the lateral ventricle**, the **body of the caudate**, and the **posterior limb of the internal capsule** (i.e. you are looking at different subparts of these structures compared to the previous slices that were rostral to the thalamus). You can still see the fornix hanging down from the **septum pellucidum** near the midline, but now this is the **body of the fornix** (because it is the part that courses over the thalamus). Again, you can see why the thalamus is such an important anatomical landmark. Again locate the **body of the corpus callosum** and the **cingulate gyrus** on the medial aspect of the slice. On the lower part of the medial aspect, below the thalamus, identify the **mammillary body**.

As always, the **lentiform nucleus** is located lateral to the internal capsule. In this slice you should be able to distinguish (from lateral to medial) the **putamen**, **lateral pallidal segment** and **medial pallidal segment**.

Now go to the temporal lobe and look for a squiggly structure, the **hippocampal formation** (**hippocampus**). The hippocampal formation lies caudal to the amygdala in the temporal lobe, so you usually don’t see it and the amygdala in the same coronal slice. However, at this transition region you may see part of the amygdala dorsal to the hippocampal formation (Fig 5.4, right side). The hippocampus lies in the floor of the **inferior horn of the lateral ventricle**, so you should always expect to see this small space lateral and dorsal to the hippocampal formation. Finally, look in the space between the temporal lobe and the internal capsule for the **optic tract**, which courses from the optic chiasm (at the level of the previous slice) to the posterior part of the thalamus.

**2.4.	Coronal Slice through the middle of the thalamus (Slice D; Image 2.4):**

This slice contains many of the same structures as the previous one. Identify the **cingulate gyrus**, **body of the corpus callosum**, **body of the lateral ventricle**, **body of the caudate**, **thalamus**, **posterior limb of the internal capsule**, **lentiform nucleus** (**globus pallidus** & **putamen**), **insula** and **lateral fissure**. The thalamus is wider in this slice than the previous one. Have you also noticed that the caudate has been getting thinner as we move posteriorly?

Look in the temporal lobe and identify the **hippocampal formation** in the floor of the **inferior horn of the lateral ventricle**. You may be able to make out the *tail of the caudate nucleus* in the roof of the inferior horn. If so, you will see that the tail of the caudate gets really thin by the time it curves down into the temporal lobe! Look between the hippocampal formation and the internal capsule for the **optic tract**, still working its way toward the posterior thalamus.

At this level, the midbrain is located ventral to the thalamus. The most ventral structure in the midbrain is the **crus cerebri** (**cerebral peduncle**). This thick white structure is the continuation of the posterior limb of the internal capsule. Dorsal to the crus cerebri, identify the **substantia nigra**, which is black in appearance because of a high density of pigmented neurons. Degeneration of these neurons, which make dopamine, causes Parkinson’s Disease. The round structure dorsal to the substantia nigra is the **red nucleus**.

**2.5.	Coronal slice through the posterior thalamus, just rostral to the posterior commissure (Slice E; Image 2.5):**

As with the previous slice, find the **cingulate gyrus**, **body of the corpus callosum**, **body of the lateral ventricle**, **body of the caudate** and **thalamus**. You probably won’t see any of the lentiform nucleus on this slice since we are caudal to it now. Go to the temporal lobe and see that the **hippocampal formation** is still there, and as always is in the floor of the **inferior horn of the lateral ventricle**. The tail of the caudate may be easier to find on this slice than the previous one.

Look dorsomedial to the hippocampal formation (toward the thalamus) for a dark nucleus shaped somewhat like a pirate’s hat. This is the **lateral geniculate body** (LGB). The LGB is the thalamic nucleus where the optic tract ends (notice that it occupies the same position as the optic tract did in the previous slices). A useful tip is that the hippocampal formation is a good landmark for both the optic tract and the LGB. Whenever you see the hippocampal formation in a coronal slice, you should always look medially and expect to see either the optic tract or the LGB (but not both, because once you get to the LGB the optic tract has ended). Look for a lighter-staining nucleus medial to the LGB. As you might expect, this is the **medial geniculate body** (MGB). The MGB is the thalamic nucleus involved in hearing (M for medial or music).

**2.6.	Coronal slice through the splenium of the corpus callosum (Slice F; Image 2.6):**

Identify the **splenium of the corpus callosum**, the **cingulate gyrus** dorsal to it, and the **trigone of the lateral ventricle** lateral to it. The trigone is the region where the body, inferior horn and posterior horn all meet. You can stick a probe into the trigone towards the occipital lobe and it will enter the *posterior horn*, which lies lateral to the calcarine sulcus. Can you use the previous slice to visualize how the body of the lateral ventricle enters the trigone rostrally and the inferior horn curves down ventrally into the temporal lobe?

Identify the **optic radiations**, they should be a thick, dark band located lateral to the trigone of the lateral ventricle. These are fibers from the LGB that are *en route* to the primary visual cortex in the occipital lobe. These fibers are not dark but white when cut lengthwise (axially).

**Part 3: Axial (Horizontal) Brain Slices**

Use the other hemisphere of your brain specimen for this part of the dissection. Start by making a horizontal slice through the body of the corpus callosum (see **Figure 2**). You don’t need to identify structures on this slice; we’re just making a flat surface to lay the next slice down. Make the remaining slices A-C, as shown in **Figure 2**. The landmarks for each slice are given as headings in the text below.

When studying the individual slices, start by placing them with the ventral surface up to best match the illustrations in the Haines Atlas and the video dissection tutorial. In some cases, the check-listed structures may be better demonstrated on the rostral surface, so don’t be afraid to look there as well. Being able to identify structures on either side of the slice is also a great way to test your knowledge of the layout of the brain!

**3.1.	Horizontal slice through the genu and splenium of the corpus callosum**

**(Slice A; Image 3.1):**

As with the coronal slices, we’ll begin with the medial and lateral aspects of the slice, which are familiar to you from your study of the intact hemisphere. After a while, you will probably be able to jump right in and start identifying the large structures at the center of the slice.

Lateral Aspect**:** Again, remember that the lateral surface of the hemisphere consists entirely of cerebral cortex. Place the slice ventral-side up and verify that the entire lateral aspect is lined by a lamina of gray matter, the cerebral cortex. That’s all there is to see here!

Medial Aspect: Rostrally, the medial surface consists of the cortex of the frontal lobe. Follow the cortex caudally to find a large white structure, the **genu of the corpus callosum**. Now go to the caudal end of the medial surface and follow the cortex of the occipital lobe rostrally to find the **splenium of the corpus callosum**. Remember from your study of the midsagittal surface of the hemisphere that the **cingulate gyrus** curves around the corpus callosum, so you see this gyrus rostral to the genu and caudal to the splenium. Look for the **thalamus** near the center of the medial aspect (rostral to the splenium of the corpus callosum). The **column of the fornix** is located anterior to the thalamus, and is attached to the genu of the corpus callosum by the **septum pellucidum**. You can verify on the intact hemisphere that you should always see the column of the fornix anterior to the thalamus on a horizontal slice.

**Where is the body of the body of the corpus callosum in relation to this slice?**

Internal Structures (**Image 3.2**): The large gray matter structure located anterior to the **thalamus** is the **head of the caudate nucleus**, and the empty space located medial to the head of the caudate and anterior to the thalamus is the **anterior horn of the lateral ventricle**. There’s that relationship between the head of the caudate and the anterior horn of the lateral ventricle again! The opening between the column of the fornix and the thalamus is the **interventricular foramen**, connecting the lateral ventricle to the third ventricle. Look for the **internal capsule**, a boomerang-shaped white structure lateral to the head of the caudate and thalamus. It is made up of fibers that descend from the cortex to the thalamus, brainstem and spinal cord, as well as fibers that ascend from the thalamus to the cerebral cortex. The **anterior limb of the internal capsule** is the part located anterior to the thalamus (and lateral to the head of the caudate). The **posterior limb of the internal capsule** is the part located lateral to the thalamus. The anterior and posterior limbs meet at the **genu of the internal capsule**, at the border between the thalamus and the head of the caudate.

Identify the **lentiform nucleus** lateral to the internal capsule. It is formed by the **putamen** (laterally) and the **globus pallidus** (medially). Note how the anterior limb of the internal capsule separates the head of the caudate from the lentiform nucleus and the posterior limb separates the thalamus from the lentiform nucleus. As you saw in the coronal slices, the lentiform nucleus is covered laterally by alternating bands of white and gray matter. From medial to lateral, these are the **external** **capsule** (white matter), **claustrum** (gray matter), **extreme** **capsule** (white matter), and **insula** (**Image 3.2**).

The space lateral to the splenium of the corpus callosum is the **trigone of the lateral ventricle**. It is connected to the anterior horn by the body of the lateral ventricle, which is located dorsal (superior) to this slice. From the trigone, the inferior horn curves down into the temporal lobe and the posterior horn extends caudally into the occipital lobe. You may be able to see the posterior horn on this slice. Look lateral to the trigone for a thick, white fiber bundle coursing from the posterior part of the thalamus toward the occipital lobe. These are the **optic radiations** (**Image 3.1**), which carry visual signals from the thalamus to the primary visual cortex. The optic radiations appear white in horizontal slices because they are cut lengthwise. They are dark in coronal sections because they are cut in cross section (see Coronal Slice F; **Image 2.6**).

**3.2.	Horizontal slice through the anterior & posterior commissures (Slice B; Image 3.3):**

This slice contains many of the same structures as the previous slide. Identify the **head of the caudate**, **thalamus**, **internal capsule** and **lentiform nucleus**. The anterior limb of the internal capsule gets very thin at this level, as we are in the region where the caudate and putamen are merging. In this slice, you can see that the lentiform nucleus consists of the **putamen**, **lateral pallidal segment** and **medial pallidal segment**. Look posterior to the head of the caudate for a distinctive band of white matter that courses laterally from the midline through the globus pallidus. This is the **anterior commissure**. It continues downward out of the plane of the slice into the temporal lobe. Just caudal to the anterior commissure on the medial aspect of the slice is a fiber tract cut in cross section, the **column of the fornix**. This nice relationship—the column of the fornix passing behind the anterior commissure—is worth reviewing once more on the medial aspect of the intact hemisphere. Now that you have seen these structures from both horizontal (axial) and vertical (coronal) point of view, review **Figure 3** to help you appreciate how these structures (the thalamus, amygdala, hippocampal formation, caudate nucleus, and fornix) are anatomically related.

**3.3.	Horizontal slice through the mammillary body & inferior colliculus**

**(Slice C; Image 3.4):**

Here you no longer see the thalamus, lentiform nucleus or internal capsule because this slice is caudal to all of these structures. In the approximate position where the thalamus was on the previous two slices, you will now see the midbrain. The most ventral structure of the midbrain is the **crus cerebri,** which are parts of the larger **cerebral peduncles**. The crus cerebri are the continuation of the posterior limb of the internal capsule and is made up of fibers from the cerebral cortex that are on their way to lower parts of the brainstem and the spinal cord. Just behind the crus cerebri is the **substantia nigra**, which is readily distinguishable by its black color. Find the round, pale-looking **red nucleus** dorsal to the substantia nigra. Notice that these are the same three structures that you can see in coronal slices through the midbrain (Coronal Slice D).

A horizontal slice at this level nicely demonstrates the layout of structures in the temporal lobe. Look for the uncus, the portion of the temporal lobe that protrudes medially just ventral to the midbrain. Do you remember looking at the uncus at the rostral end of the parahippocampal gyrus on the intact hemisphere? The large nucleus situated within the uncus is the **amygdala**. Caudal to the amygdala, locate the **hippocampal formation**. The amygdala and hippocampus are both part of the limbic system, which is important for memory and emotions. The space lateral to hippocampus is the **inferior horn of the lateral ventricle**. Follow the inferior horn rostrally to see that the amygdala is located at its rostral tip.

Find the **optic tract** coursing between the crus cerebri and the temporal lobe. It is on its way to the lateral geniculate body of the posterior thalamus. Also identify the **mammillary body** (part of the hypothalamus) on the medial aspect of the slice, medial to the crus cerebri. Finally, go anteriorly to the frontal lobe and identify the **gyrus rectus** on its medial edge.

**Checklist**

**Midsagittal Aspect of Cerebral Hemispheres:**

Corpus callosum (rostrum, genu, body, splenium)

Septum pellucidum

Fornix (body & column)

Anterior commissure

Lamina terminalis

Lateral ventricle (anterior horn & body) Caudate nucleus (head & body) Choroid plexus

Interventricular foramen (foramen of

Monro)

Third ventricle

Thalamus

Massa intermedia

Hypothalamus

Optic chiasm

Infundibulum

Mammillary body

Pineal body

Posterior commissure

Cerebral aqueduct

Superior Colliculus

Inferior Colliculus

Midbrain tegmentum

Basilar Pons

Pontine tegmentum

Fourth ventricle

Cerebellum

Superior medullary velum

Medulla

**Coronal Brain Slices:**

Lateral fissure

Insula

Cingulate gyrus

Corpus callosum (body & splenium)

Septum pellucidum

Lateral ventricle (anterior horn, body, trigone & Inferior horn)

Caudate nucleus (head & body)

Internal capsule (anterior limb & posterior limb)

External capsule

Claustrum

Extreme capsule

Lentiform Nucleus: Putamen & Globus pallidus (lateral pallidal segment &

medial pallidal segment)

Anterior commissure Fornix (body & column)

Amygdala

Thalamus

Mammillary body

Hippocampal formation

Crus cerebri (cerebral peduncle)

Substantia nigra

Red nucleus

Optic Tract

Lateral geniculate body (LGB)

Medial geniculate body (MGB)

Optic radiations

**Axial Brain Slices:**

Genu of corpus callosum

Splenium of corpus callosum

Cingulate gyrus

Thalamus

Column of fornix

Septum pellucidum

Anterior horn of lateral ventricle

Interventricular foramen (F. of Monro)

Head of caudate nucleus

Lentiform Nucleus: Putamen & Globus Pallidus (lateral pallidal segment & medial pallidal segment)

Insula

Internal capsule (anterior limb, genu & posterior limb)

External capsule

Claustrum

Extreme capsule

Trigone of lateral ventricle

Optic radiations

Anterior commissure

Amygdala

Hippocampal formation

Inferior horn of lateral ventricle

Optic tract

Mammillary body

Crus cerebri (cerebral peduncle)

Substantia nigra

Red nucleus

Gyrus rectus

**Images**

**Tools for Dissection:**
