# Supplementary material for: A Multimedia Educational Module for Teaching Early Medical Neuroanatomy
Source: MedEdPORTAL. 2020 Mar 6;16:10885. doi: 10.15766/mep_2374-8265.10885 (PMC7067615; doi:10.15766/mep_2374-8265.10885)
Supplement: Supplementary file 1 — A. Predissection Questionnaire.docx B. References for Predissection Questionnaire.docx C. External Topography Dissection Instructions.docx D. Internal Topography Dissection Instructions.docx E. External Topography Dissection Video Tutorial.mp4 F. Internal Topography Dissection Video Tutorial.mp4 G. External Topography Review Slides.pptx H. Internal Topography Review Slides.pptx I. External-Internal Survey.docx [file mep-16-10885-s001.zip › I. External-Internal Survey.docx]

The following questions refer to the resources for LAB #1: **EXTERNAL TOPOGRAPHY OF THE BRAIN** (Video & Interactive PowerPoint)

1a. How many times did you view each of the following resources PRIOR TO the EXTERNAL TOPOGRAPHY OF THE BRAIN laboratory session?

|  | **0** | **1** | **2 to 4** | **5 or more** |
| --- | --- | --- | --- | --- |
| **VIDEO** |  |  |  |  |
| **INTERACTIVE POWERPOINT REVIEW** |  |  |  |  |

1b. How many times did you view the following resources AFTER THE LABORATORY SESSION in preparation for the exam?

|  | **0** | **1** | **2 to 4** | **5 or more** |
| --- | --- | --- | --- | --- |
| **VIDEO** |  |  |  |  |
| **INTERACTIVE POWERPOINT REVIEW** |  |  |  |  |
| **PRESERVED BRAIN SPECIMEN** |  |  |  |  |

1c. How helpful were the following resources in preparing you to **perform the laboratory exercise**?

|  | **Extremely helpful** | **Very helpful** | **Somewhat helpful** | **Not so helpful** | **Not at all helpful** | **Did not Use** |
| --- | --- | --- | --- | --- | --- | --- |
| **VIDEO** |  |  |  |  |  |  |
| **INTERACTIVE POWERPOINT REVIEW** |  |  |  |  |  |  |

1d. How helpful were the following resources as aids for **learning the relevant anatomy**?

|  | **Extremely helpful** | **Very helpful** | **Somewhat helpful** | **Not so helpful** | **Not at all helpful** | **Did not Use** |
| --- | --- | --- | --- | --- | --- | --- |
| **VIDEO** |  |  |  |  |  |  |
| **INTERACTIVE POWERPOINT REVIEW** |  |  |  |  |  |  |
| **CADAVERIC BRAIN SPECIMEN** |  |  |  |  |  |  |

(SURVEY CONTINUES ON THE OPPOSITE SIDE OF THE PAGE)

The following questions refer to the resources for LAB #2: **INTERNAL TOPOGRAPHY OF THE BRAIN**. (Video & Interactive PowerPoint)

1a. How many times did you view each of the following resources PRIOR TO the INTERNAL TOPOGRAPHY OF THE BRAIN laboratory session?

|  | **0** | **1** | **2 to 4** | **5 or more** |
| --- | --- | --- | --- | --- |
| **VIDEO** |  |  |  |  |
| **INTERACTIVE POWERPOINT REVIEW** |  |  |  |  |

1b. How many times did you view the following resources AFTER THE LABORATORY SESSION in preparation for the exam?

|  | **0** | **1** | **2 to 4** | **5 or more** |
| --- | --- | --- | --- | --- |
| **VIDEO** |  |  |  |  |
| **INTERACTIVE POWERPOINT REVIEW** |  |  |  |  |
| **PRESERVED BRAIN SPECIMEN** |  |  |  |  |

1c. How helpful were the following resources in preparing you to **perform the laboratory exercise**?

|  | **Extremely helpful** | **Very helpful** | **Somewhat helpful** | **Not so helpful** | **Not at all helpful** | **Did not Use** |
| --- | --- | --- | --- | --- | --- | --- |
| **VIDEO** |  |  |  |  |  |  |
| **INTERACTIVE POWERPOINT REVIEW** |  |  |  |  |  |  |

1d. How helpful were the following resources as aids for **learning the relevant anatomy**?

|  | **Extremely helpful** | **Very helpful** | **Somewhat helpful** | **Not so helpful** | **Not at all helpful** | **Did not Use** |
| --- | --- | --- | --- | --- | --- | --- |
| **VIDEO** |  |  |  |  |  |  |
| **INTERACTIVE POWERPOINT REVIEW** |  |  |  |  |  |  |
| **CADAVERIC BRAIN SPECIMEN** |  |  |  |  |  |  |
